# It takes biking to learn: Physical activity improves learning a second language.

**DOI:** 10.1371/journal.pone.0177624

**Published:** 2017-05-18

**Authors:** Fengqin Liu, Simone Sulpizio, Suchada Kornpetpanee, Remo Job

**Affiliations:** 1 Dali University, Dali, China; 2 Vita-Salute San Raffaele University, Milano, Italy; 3 Fondazione Marica De Vincenzi onlus, Rovereto (TN), Italy; 4 Burapha University, Bangsaen, Thailand; 5 University of Trento, Trento, Italy; University of Zurich, SWITZERLAND

## Abstract

Recent studies have shown that concurrent physical activity enhances learning a completely unfamiliar L2 vocabulary as compared to learning it in a static condition. In this paper we report a study whose aim is twofold: to test for possible positive effects of physical activity when L2 learning has already reached some level of proficiency, and to test whether the assumed better performance when engaged in physical activity is limited to the linguistic level probed at training (i.e. L2 vocabulary tested by means of a Word-Picture Verification task), or whether it extends also to the sentence level (which was tested by means of a Sentence Semantic Judgment Task). The results show that Chinese speakers with basic knowledge of English benefited from physical activity while learning a set of new words. Furthermore, their better performance emerged also at the sentential level, as shown by their performance in a Semantic Judgment task. Finally, an interesting temporal asymmetry between the lexical and the sentential level emerges, with the difference between the experimental and control group emerging from the 1^st^ testing session at the lexical level but after several weeks at the sentential level.

## Introduction

Learning words requires acquiring several types of information about each item, e.g., conceptual, semantic, morphological, phonological information, as well as information about relations among words. In the native language, such process develops over time, is tacit, and is highly efficient. For example, a single encounter with a word, given proper contextual constraints, is sufficient for learning some aspects, e.g., the meaning of the word [1]. It interacts with conceptual development, although how the two processes affect each other is still debated (for reviews see, e.g. [2, 3]).

Learning a second language (L2) after the native one presents with a novel situation, as the bulk of the individual’s conceptual representation has been already developed. This leaves the possibility that the learner either constructs a new conceptual representation for the L2 word, or that he/she links the new lexical form to an existing concept that is already associated to the corresponding L1 word (see, e.g. [4, 5]). The available evidence on this issue is mixed, and one of the pivotal questions addressed in the field is how the mapping between the L2 word and the concept on the one hand, and between L2 and L1 words on the other takes place.

Several studies have addressed this issue by comparing two ways to mediate between the lexical level and the conceptual level, i.e. through words and through pictures, on the basis of the assumption that different factors affect the mapping into concepts in the two conditions (see, e.g. [6, 7]).

The level of knowledge of the language attained, and the efficacy in the use of the language, which converge in defining fluency, plays a crucial role in the strategy actually adopted by individuals in accessing the conceptual system from words (e.g. [8]). The Revised Hierarchical Model by Kroll and Steward [9] nicely captures this state of affaires by postulating a langue-independent conceptual system and two language-specific lexica whose inter-relations are shaped by language learning and use. Specifically, when learning L1 and L2 is asynchronous, in the initial stages of L2 vocabulary acquisition words in L2 are mapped to words in L1 rather than directly mapped to the corresponding concepts (note that throughout the paper we will use the term learning and acquisition to refer to the processes mediating the mapping between the orthographic/phonological forms of words and their lexical representation). The learning of L2 words is thus mediated by L1 words, and access to the conceptual representation is indirect, via L1. At later stages, with increased fluency in L2, words in L2 are mapped directly to the corresponding concepts, without the need to rely on L1 words. The mappings and the links among the components of the model should not be considered univocal, as there is empirical data showing that processing a L2 word activates to some degrees similar L1 words (in addition to similar L2 words) as well as the corresponding concepts (e.g. [10, 11]). Thus, it is more appropriate to talk about connections among the components that vary in strength and/or distance and that are dynamically affected by fluency, as well as mode of learning and other relevant factors (such as cognate status, see e.g. [12]).

Recently, some studies investigated the issue of the role of physical activity in vocabulary learning taking, as starting point, the empirical data suggesting a positive effect of physical activity in cognitive processing, in particular memory processes. Much empirical evidence is available on the effects of physical activity on cognition. Several animal studies have shown a strong influence of physical activity on synaptic plasticity, in particular on the genesis of new neurons [13–16], and on the release of several neurotrophic factors [17, 18]. Also studies with humans have addressed the problem, both at the neural and at the behavioral level (e.g. [19–23]).

As for vocabulary learning, two studies are especially relevant. Winter and colleagues [24] assessed the ability of healthy adult German males to learn a novel vocabulary under three conditions: a) after high intensity anaerobic sprints; b) after 40 minutes of low intensity aerobic running; or c) after 15 minutes being sedentary. The vocabulary learning comprised two phases, i.e. a training phase and a transfer phase. The training phase took place after the physical activity and the collection of blood sample to test for neurotrophic factors. During training, participants were presented with stimuli pairs comprising a spoken pseudoword and a picture of an object and had to decide if the pairing was correct (i.e. consistent with the association stipulated at the beginning of the experiment) or not (see [25] for details). In the transfer phase that took place immediately after the training session and again after a week and after 6–8 month, the visually depicted objects were substituted by the corresponding German names. Participants had to decide whether the pseudoword and the object spoken name pairing were correct or not.

Two results are especially important for the present discussion. First, vocabulary learning was 20% faster when it took place after the high intensity exercise compared to both the low intensity exercise and the sedentary condition. Second, the high intensity exercise elicited the strongest increase in the level of brain-derived neurothropic factor and of catecholomines (see also [26]). The authors interpret these data as showing that exercise improves learning of, and helps long-term memory for, new vocabulary items.

Schmidt-Kassow and colleagues [27] addressed the issue of vocabulary learning and physical exercise by looking at the long-term effect of regular physical activity (in contrast to a single bout), but also investigating whether physical activity during learning also accelerates the learning process. In their study, two conditions were considered: Spinning, involving simultaneous bicycling and learning, and passive, with learning without physical activity. Participants were German native speaker and were asked to learn 80 French words, half nouns and half verbs, in a period of three weeks (with three learning sessions per week). Two indexes of learning were used. First, at the end of each week participants performed a vocabulary test in which they were asked to write down the German translation of the spoken French words. In addition to these behavioral measures, electrophysiological responses were recoded during a cross-language priming experiment run prior to and after the three-week training. Participants were presented with spoken French-German and German-French word-word and word-pseudoword pairs and performed a lexical decision task on the second item of each pair. Some of the French-German word-word pairs were translation of the same concept in the two languages (e.g. chien-Hund, 'dog-dog') while the others were unrelated (e.g. gateau-Hund, 'cake-dog') and such asymmetry is sensitive to the N400 component of ERPs, considered a signature of semantic processing. Two findings are of interest. First, at the behavioral level, performance in the spinning condition was better than performance in the passive condition in all three testing session. Second, a larger N400 effect was found at the post-training session for the spinning group compared to the passive group, while no difference for the two groups was found at the pre-training session. As the N400 is considered a signature of semantic processing, among other aspects such as expectancy and prediction, we may conclude that sensitive to semantic relationship between items in the two languages was acquired very early. A recent study by the same research group confirms that physical activity benefits L2 learning when they are simultaneous [28].

The studies by Schmidt-Kassow et al. [27, 28] are particularly interesting as they use a dual-task condition. By and large, allocation of attention to the two tasks hinders performance in one or both tasks when compared to a single task condition (but see [29]). This is certainly true for most combination of concurrent tasks, but the literature is not univocal when a cognitive task is performed concurrently with a physical exercise: Two recent meta-analyses reported either a decrease in performance during the first 20 minutes of physical exercise on a variety of cognitive tasks [30] or a positive, albeit small, effect throughout the session [31]. Both studies reported positive effects when the cognitive task followed the physical exercise. None of the studies in the meta-analyses addressed vocabulary learning.

In both Winter and colleagues’ [24] and Schmidt-Kassow and colleagues’ [27, 28] studies participants (a) were totally unfamiliar with the L2 they were presented with, and this leaves open the possibility that the positive effect of physical exercise is limited to the very initial stages of learning L2 when fluency is very limited or non-existent; (b) were trained on, and tested for, the same linguistic level, i.e. the lexicon, and this is silent as to weather learning the L2 vocabulary allows for the productive use of the learned words in a sentential context; (c) showed an advantage of the learning condition associated to physical activity irrespective of the successive [24] or concurrent performance on the two tasks [27, 28], and this motivates a conceptual replication of the double-task situation—which has yield less univocal findings according to the available meta-analyses (cf. [30, 31]).

## The present study

The aim of the present study is to further investigate the influence of physical activity on L2 learning. To this end, an experiment was run with Chinese-English bilingual learners who were presented with picture-word pairs in the learning phase, and were required to perform both a lexical verification task and a sentence semantic judgment task in the testing phase. The main motivation for the study is twofold.

First, to test whether the assumed better performance (faster RTs, higher accuracy) of the students engaged in physical activity is limited to the linguistic level probed at training (i.e. L2 vocabulary), or whether it extends also to the level of sentence processing as indexed by a sentence judgment task. According to several studies (e.g. [32]) a picture-based L2 vocabulary teaching method favours the establishment of direct links between the L2 lexicon and the conceptual system, downplaying the role of the L1 lexicon as a translation device; in turn, this should allow L2 words to benefit from the rich conceptual representation already available to the learner. In order to boost the semantic analysis of L2 words we used a picture-based method, predicting that such analysis may enhance semantic processing of the relationship among words, thus enhancing sentence level processing. Thus, we predict that students performing the dual task will perform better than students learning in a sedentary condition also at the sentence level. This issue has never been addressed before, neither in connection with physical activity nor when addressing semantic processing in L2 vocabulary learning.

Second to test whether the possible positive effects of physical activity in L2 learning are present when learners are not completely unfamiliar with the target L2 but have some, albeit incomplete, knowledge of it. According to the Revised Hierarchical Model [9], there is a shift over time on the reliance on L1 words to access concept when using L2 words: beginning learners rely more on L1 words than advanced learners, while for fluent L2 speakers there is direct access to the conceptual system. Differences along the dimension of mastering a L2 language are thus important and may be akin to different mapping strategies. Since the participants tested by Smith-Kassow et al. ([27, 28]) had no knowledge of the foreign language they were taught it is worth, both theoretically and practically, investigating whether physical activity may benefit more advanced L2 learners.

### Participants

The present research was conducted in accordance with the ethical standards laid down in the 1964 Declaration of Helsinki. All participants provided consent before participating. The research has been approved by the Ethical Committee of Burapha University.

Forty-two participants were asked to participate in the study, but two of them were removed from the sample because they did not complete the full experimental procedure (one did not complete the training session and the other one did not complete the test session). The final sample was composed of 40 right-handed late Chinese-English L2-learners undergraduate students at Dali University. Participants were healthy, with no history of major physical trauma, cardiovascular and respiratory disease, and neurological and psychiatric disease; they gave their written consent and were randomly assigned to one of two conditions: simultaneous physical activity during learning (20 participants: Experimental group), and static or conventional learning (20 participants: Control group). The two groups were balanced for gender and age of the participants (Experimental: 10 males (mean age 19.7 years), 10 female (20.3 years); Control: 11 males (20.3 years), 9 females (20.2 years)). For all participants, L2 proficiency and aerobic fitness were tested before the experiment. The College English Test Band 4 (CET– 4, see [33]) was used to assess the participants’ proficiency level for English. In order to have a balanced group of beginning learners only participants who scored between 290–350 out of the possible maximum score of 710 were selected. The experimental and the control groups did not differ for English proficiency (t (38) < 1, p >.3). For physical fitness, the participants were tested on the Queens College Step (e.g., [34, 35]) and for each the VO2max, a measure of a person's aerobic fitness, was estimated using the McArdle et al. 1972’s formula [36]. In order to be included in the study, participants’ score on the VO2 max had to be at least 60 (ml/kg/min.) for males and at least 42 (ml/kg/min.) for females. A final check showed that the experimental and the control group did not differ on age (20.00 vs 20.25, t (38) < 1, p >.3), English level (mean score at the CET-4: 328.8 vs 326.4 for the experimental and the control group, respectively, t (38) <1, p >.3); fitness level (mean VO2max level: 52.1 vs. 51.4 for experimental and control group, respectively, t (38) <1, p >.3). Moreover, since participants were randomly selected from the general pool of students having passed the Chinese NCEE (National College Entrance Examination) their cognitive and cultural competency are comparable. At the beginning of the study, all of the participants were instructed to avoid changes in their standard physical activity level for the duration of the experiment.

### Material

Forty English written words and the corresponding black-and-white pictures (UCSD, CRL database) were selected from the categories of food, animals, objects, and professions, avoiding as much as possible visually similar picture in order to minimize visual, as opposed to semantic, effects [7]. All words were unknown to the participants before the experiment. Words were also auditorily recorded. Following Tonzar, Lotto, and Job’s [37] procedure, words and pictures were used both in the learning phase and in the test phase (see also [32]).

Four verbs were also selected in order to construct 20 semantically well-formed and 20 semantically ill-formed sentences to be used in the test phase. The verbs were: *eat*, *follow*, *push*, and *break*, and were selected in order to allow for animate and inanimate concepts to be in subject and object position in the sentence, making it possible to have semantically well formed and semantically ill-formed sentences by varying the order of animate and inanimate concepts. The verbs were presented in their English form, with the corresponding Chinese translation, to the participants who were asked to memorize them in order to perform a Semantic Judgment task on the set of well- and ill-formed sentences. Acceptability ratings on a 1–5 Likert-type scale (5 = fully acceptable) collected from a sample of Canadian English native speakers (50 participants, 13 males, *mean age*: 20.27, *sd*: 1.77) confirmed that the two types of sentences differed significantly on the semantic acceptability dimension (well-formed sentences: *mean*: 4.05, *sd*: 0.58; ill-formed sentences: *mean*: 1.57, *sd*: 0.42, t(49) = 30.33, p < .001).

The experiment was run by means of the DMDX software (version 4.2.2.0 [38]).

### Procedure

#### The learning phase

Participants were presented sequentially, via a projector, the list of pictures along with their written and spoken names. Each picture-name pair was displayed for 5 sec. The 40 picture-name pairs were presented in 2 blocks of 20 pairs each, with a short interval between the two blocks, for three consecutive times so that each participant was exposed to the same picture-name three times in each session. In each block, order of presentation was randomized.

The learning phase took place in a quiet lab room and each participant was tested individually. There were 8 learning sessions held one week apart. Participants were randomly assigned to either the experimental or the control condition.

In the experimental condition participants were required to ride a bicycle during stimulus presentation. The ergometer bicycle was equipped with an adjustable workload (resistance) and a meter that provided a visual indication of cadence (pedaling rate). Participants were required to wear a Polar Edge heart rate monitor–able to provide a second-by-second digital record of heart rate throughout the experiment–in order to check and control for the intensity of the exercise. The aerobic work started at a level of exercise that requires the heart to beat at 60% of an individual’s maximum heart rate [39]. In this study the estimate of maximum heart rate (in beats per minute, bpm) was '220 minus the participant’s age’. Twenty minutes prior to the learning phase, the participant began pedaling at a cadence of 60 rpm for two minutes as warm up activity. The resistance of the ergometer was adjusted via the Polar Edge heart rate monitor. As the stimuli were presented, the participant continued to pedal at this rate throughout the learning phase.

In the control condition (static or conventional learning) the same presentation parameters and procedure of the learning phase were used, but the participant sat on a chair in front of a table.

#### The test phase

In order to quantify learning, participants were asked to perform 9 testing sessions. In each session, two tasks were employed: a Word-Picture Verification task and a Semantic Judgment task.

The first 8 test-sessions were performed at the end of each of the 8 learning phases while the last was held one month after the last test session.

In each test session the first task was the Word-Picture Verification and the second the Semantic Judgment. Prior to the test, participants were familiarized with the tasks by presenting 2 training blocks with 10 trials in each block.

*Word–Picture Verification tasks*. Twenty “old” and 20 “new” picture-name pairs were used in each test session. “Old” pairs refer to the picture-word combinations presented during learning, i.e. correct combinations (e.g. the picture of a queen and the word “queen”) while “new” pairs were constructed by rearranging pictures and words presents during the learning phase in such a way as to give rise to incongruent pairs (e.g. the picture of a walnut with the word camel). Each picture and each word appeared only once during each test session, and order of presentation was randomized. On each trial, a fixation point (+) was displayed for 500ms and was followed by the picture-word pair (with the picture positioned above the word) displayed for 1500 ms. (1200 ms from trial 5 onward). The response was given by pressing one of two keys–“Z” (for congruent pairings) and “M” (for incongruent pairings)–and a feedback was provided by showing the response time on the screen. If the responses took more than 2000 ms (1500 ms from trial 5 onward) no response was recorded and the computer automatically moved on to the next stimulus.

*Sentence Semantic Judgment tasks*. Twenty semantically well-formed and 20 ill-formed sentences were constructed. The sentences were obtained by combining two of the words of the experimental list (one in subject position and one in object position) with one of the four verbs participants were asked to memorize. Each sentence had the form “Determiner + Noun + Verb + Determiner + Noun”. In the semantically well-formed sentences the relationships between the elements of the sentences were felicitous (e.g. the dentist eats the peas) while in the semantically ill-formed sentences they were pragmatically infelicitous (e.g. the camel breaks the nurse). Each sentence appeared only once during each test session, and order of presentation was randomized. On each trial, a fixation point (+) was displayed for 500 ms and was followed by the sentence (written on a single line) displayed for 5000 ms (4000 ms from trial 5 onward). The onset of the sentence triggered the time counter that was stopped by the participant’s response. The response was given by pressing one of two keys–“Z” (for congruent pairings) and “M” (for incongruent pairings)–and a feedback was provided by showing the response time on the screen. If the responses took more than 5000 ms (4000 ms from trial 5 onward) no response was recorded and the computer automatically moved on to the next stimulus.

## Results

### Word-Picture verification task

**Fig 1:**
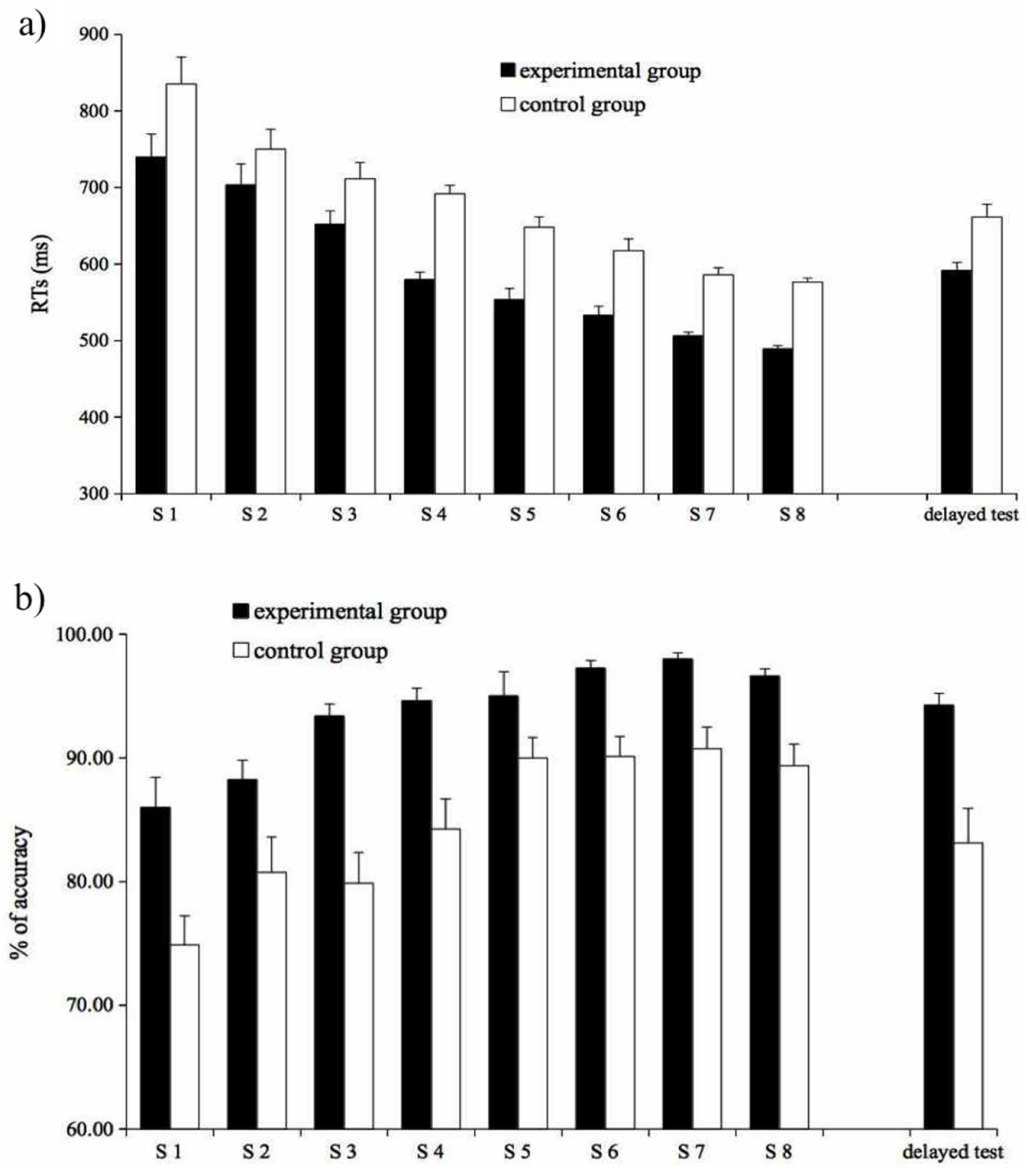
Word-Picture verification task. Mean RTs (1a) and percentage of accuracy (1b) by group for each test session (S1-S8) and for the delayed test. Vertical bars indicate standard error. Note that y-axes do not start from 0.

The Reaction Times (RTs) of correct responses and Percentages of Correct Responses (overall 89.32% of all data points) are reported in Fig 1A and 1B, respectively.

Data were analyzed by means of mixed-effects models [40, 41]. The models were fitted in R software using the lmerTest package [42].

A linear mixed-effects model was run on the mean RTs of correct responses with group (experimental vs. control) as dichotomous factor (with “control group” as baseline level of the factor) and test session (session 1 to 8) as continuous predictor; participants were treated as random factor. The model showed a main effect of group (β = -73.76, st. err = 22.92, t = -3.21, p = .001): the participants in the experimental group (M = 594, SD = 54.98) were faster in verifying the words in L2 than participants in the control group (M = 677, SD = 56.20); the main effect of test session was also significant (β = -35.21, st.err. = 2.31, t = -15.24, p < .001), with response time decreasing over the 8 sessions. Noticeable, the interaction between the groups and the test sessions was not significant (t < 1, p >.5).

A logistic mixed-effects model was run on proportion of correct responses with group (experimental vs. control) as dichotomous factor (with “control group” as baseline level of the factor) and test session (session 1 to 8) as continuous predictor; participants were treated as random factor. The model showed a significant effect of group (β = 0.13, st. err. = 0.05, z = 2.42, p = 0.01), with participants in the experimental group (M = 93.64, SD = 3.42) being more accurate than participants in the control group (M = 85.00, SD = 8.05), and a significant main effect of accuracy rate for test sessions (0.02, st. err. = 0.01, z = 3.285 p = 0.001), with errors decreasing over the 8 sessions. The interaction was not significant (z < 1, p >.4), paralleling the pattern of RTs.

### Semantic judgment task

**Fig 2:**
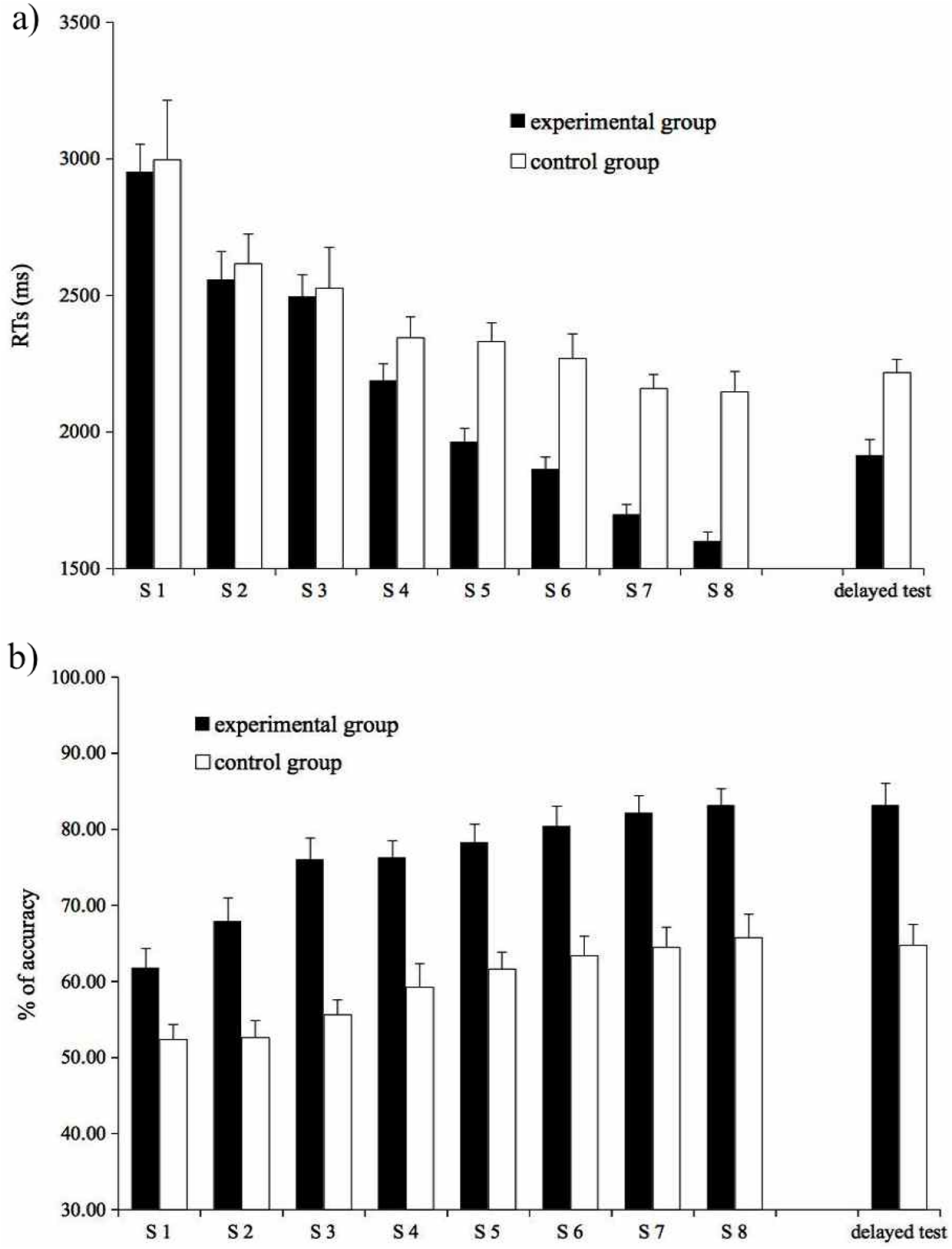
Semantic judgment task. Mean RTs (2a) and percentage of accuracy (2b) by group for each test session (S1-S8) and for the delayed test. Vertical bars indicate standard error. Note that y-axes do not start from 0.

The Reaction Times of correct responses and Percentages of Correct Responses (overall 67.55% of all data points) are reported in Fig 2A and 2B, respectively.

Mean RTs of correct responses were analyzed by means of a linear mixed-effects model with group (experimental vs. control) as dichotomous factor (with “control group” as baseline level of the factor) and test session (session 1 to 8) as continuous predictor, and participants as random factor. The results showed a significant main effect of test sessions (β = -107.34, st. err. = 11.54, t = -9.29, p < .001), with response time decreasing over the 8 sessions. More interestingly, the interaction between the groups and the test sessions was significant (t = -81.69, st. err. = 16.33, t = -5.004, p < .001) indicating that the difference between the experimental and control group emerged and grew along the sections. Specifically, the experimental group sensibly speeded up response times with respect to the control group. The response times of the control group continued to decrease too, but at a lower rate. This pattern indicates a strong advantage in L2 learning during physical activity at the sentence level.

Response accuracy was analyzed by means of a logistic mixed-effects model, with proportion of correct responses as dependent variable, and group (experimental vs. control; dichotomous factor with “control group” as baseline level of the factor) and test session (session 1 to 8; continuous variable) as predictors; participants were treated as random factor. The model showed both a significant effect of group (β = 0.23, st. err. = 0.07, z = 3.33, p < .001), with the experimental group (M = 75.72, SD = 9.39) being more accurate that the control group (M = 59.39, SD = 8.56) in deciding whether a sentence is correct or not, and a significant effect of session (β = 0.03, 0.01, z = 3.977, p < .001), with accuracy rate increasing across sessions. The interaction between group and test session was not significant (z < 1, p >.8).

### Delayed testing

In order to verify possible long-lasting effect in L2 vocabulary learning, a delayed test was conducted 4 weeks after the last learning-and-test session. The delayed test was the same as the tests at the end of each session. Mixed-effects models were performed on the data from the Delayed test compared with the data from the test performed at the end of the 8^th^ (and last) learning session. The models were run with Group (experimental vs. control, with “control as baseline level) and Test session (Test at session 8 vs. Delayed test, with “Test at session 8” as baseline level) as fixed factors, and participants as random factor.

The RTs and the accuracy rates of the delayed test for the Word-Picture Verification task are reported in Fig 1A and 1B (the two right-most bars) and those of the Sentence Semantic Judgment task are reported in Fig 2A and 2B, respectively (the two right-most bars).

#### Word-Picture verification task

The model on RTs showed a significant effect of group (β **=** -87.45, st. err. = 14.85, t = -5.888, p < .001), with the experimental group (M = 540, SD = 63.13) being faster in verifying the word than the control group (M = 619, SD = 69.82), and a significant effect of test sessions (β **=** 84.95, st. err. = 12.50, t = 6.798, p < .001), due to the longer RTs in the delayed test than in the test performed in the 8^th^ session (626 ms vs. 532 ms). The interaction between test sessions and groups was not significant (F (1, 38) = 1.03, p = .3).

The logistic model on proportion of correct responses showed no significant effects (group: z = 1.08, p >.2; test session: z < 1, p .3, group x test session: z < 1, p >.6).

#### Sentence semantic judgment task

The model on RTs showed a main effect of group (β **=** -547.65, st.err = 79.16, t = -6.918, p < .001). The interaction was also significant (β = 243.65, st. err. = 111.95, t = 2.17, p = .03), showing that the difference between the two groups was significant in both the last training session (difference: 548 ms, p < .001) and the test session, but larger in the former than in the latter (difference: 304 ms, p = .003). The main effect of Test session was not significant (t < 1, p >.3).

The logistic model on proportion of correct responses showed a main effect of group (β = 0.23, st. err. = 0.07, z = 3.05, p = .002), with the experimental group (M = 83.12, SD = .11.43) performing better than the control group (M = 65.25, SD = 12.94). No further effect reached significance (test session: z <1, p >.8; experimental group x test session: z <1, p >.8).

## Discussion

The results of the study are clear-cut: learning a foreign vocabulary while performing a concurrent physical activity yields better performance than learning the same vocabulary while being in a static situation. Such finding is qualified by the associated results that (a) the improved performance in the test phase is not limited to the linguistic level trained in the learning phase, i.e. the acquisition of the L2 vocabulary, but extends to the level of sentence processing as well, since participants in the physical activity group performed better than the control group also in the sentence judgment task and (b) the advantage of the physical activity group in both lexical and sentential processing seems to be long-lasting, as shown by the results of the test performed after a month from the last learning session without intervening learning trials.

This pattern confirms previous studies that have shown that exercise positively affects cognition in several ways (e.g. [43, 44]) e.g. by slowing down age-related cognitive decline, by allowing efficient allocation of attention, and by improving executive control functions. They are also consistent with the studies that have focused on a specific aspect of cognition, verbal learning (e.g. [27, 28]), and have shown a positive effect of physical intervention on vocabulary learning.

Several explanatory hypotheses have been advanced to account for the relationship between physical activity and cognition, and several studies are now available that make finer distinction as to what type of physical activity (e.g. moderate, continuous, single burst) and what aspects of cognition (e.g. working memory, executive function, verbal learning) are involved. By and large, physical activity is supposed on the one hand to favor synaptic plasticity and on the other hand to increase the availability of specific neurotrophic substances in the brain, such as BDNF (Brain-Derived Neurotrophic Factor) [45, 46], that facilitate learning.

The data here reported extend previous findings in two ways.

First, participants showed effects of physical activity when re-tested after four weeks without intervening trials. This pattern rules out the possibility that the effect of physical activity may be due to a general arousal level that boosts immediate performance rather than prompting true learning with consequences at the level of memory encoding. Thus, from this pattern we may infer that it is indeed the process of learning L2 that is affected by physical activity, and that this effect is long-lasting. As an aside, we may wonder if the effect reported for the experimental group is somewhat underestimated (or, conversely, the effect of the control group is overestimated). It is known, in fact, that memory performance is sensitive to the so-called “context” effect: when the context of encoding and the context of retrieval are the same, as e.g. when a list of words is encoded under water and the recall occurs under water, performance is better compared to the situation in which the two contexts differ, e.g. a list of words is encoded under water and the recall occurs on land [47]. This effect is stronger for free recall but it is also present for recognition. Since in the present study the encoding and the verification phases were quite similar for the control group (static at encoding and static at verification) but differed markedly for the experimental group (moving at encoding but static at verification), the better performance of the latter group is even more noteworthy.

The pattern of results confirms that in a dual task condition in which L2 learning and physical activity are concurrent there is a positive effect of the physical activity on learning. There are, to our knowledge, no studies on L2 learning in a dual-task condition apart from those by Schmidt-Kassow et al. [27–28], and our results are consistent with theirs. The pattern we report is compatible with one of two possible accounts. First, the physical activity does not constitute a competitive task for L2 learning at the cognitive level, while providing a benefit at the neurophysiological level. Recently, Song and Bedard [29] have shown that when in a dual-task situation attention is high (or none) to both the primary and secondary task performance was better than when only one of the two task required attentional resources. We would like to propose that in our dual-task setting both tasks required high attentional resources. Second, the physical activity does indeed compete for attentional and cognitive resources (e.g. working memory) with the L2 learning, but the benefit at the neurophysiological level compensate, and indeed exceed, the limitation brought about the double task. The available data does not allow selecting between the two alternatives, and speculations on this issue are premature. However, disentangling the effects of task combination may be worth pursuing to better understand the underlying mechanisms.

A further finding deserving discussion is the fact that physical activity exerts its effect when participants have already been exposed to L2 for some time and already know some aspects of the language. In all the previous studies using this paradigm participants were totally unfamiliar with L2 they were exposed to during the experiment, while participants in the present study were university students who had passed the CET-4 test, a test routinely used to examine the English proficiency of undergraduate students and postgraduate students in China. On the basis of their scores, they can be classified as beginners. Our data can be interpreted as evidence for an effect of physical activity not only during L1-mediated access to conceptual representations, in terms of The Revised Hierarchical Model [9], but also when learners start to use L2-mediated access. That is to say, they might facilitate and/or temporally speed up the strengthening of the links between L2 words and concepts.

The finding that physical activity benefit not only people unfamiliar with L2 but also people who already knows an L2 at basic level has implication for learning-supportive environments, such as schools and rehabilitation centers, since it suggests finding ways to integrate physical activity and learning in order to improve the latter. Such integration may not be easy to plan and to implement but the data here reported show that the temporal contiguity between physical activity and L2 learning may be effective and beneficial.

The superiority of the physical activity group emerged both in the Picture-Word Verification task and in the Sentence Semantic Judgment task. In the former case, the task could be performed on the basis of a memory search (see e.g. [27]). However, the fact that the better performance of the physical activity group generalizes to the sentential levels is a novel finding and this allows qualifying the effect. One of the possible reasons for the effect is that it is “simply” the results of the participants’ being temporarily better in using their attentional resources in the experimental condition as a consequences of the sense of novelty and/or surprise: While it is common and conventional to learn a subject matter sitting at a desk, in a sedentary situation, it is quite uncommon, and maybe surprising, to learn it while pedaling a bicycle. Thus, the experimental group could have devoted more attention to the picture-word association during learning and hence performed better during the vocabulary verification test. However, the advantage of the experimental group generalizes to the sentential level that requires computing the grammatical as well as the meaning relations among the elements of the sentence and this runs against an account in terms of rote memorization of the picture-word pairs. Interestingly, the sentential level provides a further cue that physical activity in our study did not promote (only) rote memorization of the stimuli. While RTs in the Picture-Word Verification task show an advantage for the physical activity group from Session 1 onward, such advantage in the Sentence Semantic Judgment task emerges after the 4^th^ Session.

What are the reasons for this asymmetry between the two tasks in relation to the effect of physical activity on cognition? One possible reason is that physical activity affects some processes (e.g. memory encoding, memory retrieval) but less so other processes (e.g. decision making). This would account for the dissociation by postulating that the two tasks differ in systematic ways with respect to the critical processes. Another possible reason has to do with the easy-vs-difficult-to-integrate dimension. That is to say, it may be possible that physical activity is more apt to affect the encoding and learning of simple, easy material, but has less effect on difficult material, and this would account for the earlier advantage for the lexical, single word level than the sentential, more complex level. A hint that may be the case comes from perusal of both Schmidt-Kassow et al.’s [27, 28] studies, from which it appears that the effect of physical activity is quite precocious. While direct comparisons are not easy as the studies differ on a number of dimensions, e.g. the number of repetition of stimuli, the data reported point to an early onset of the effect, in all those studies the benefit for vocabulary learning is quite early. In particular, the Schmidt-Kassow et al.’s [28] study a significant difference between the condition with and without physical activity is reported already on the testing performed on day one.

However, we may also hypothesize that, because of the knowledge about L2 our participants already possess, and because of the task we used, that maximizes conceptual encoding, participants relied to a large degree on direct links between L2 words and concepts, rather then using the mediation of L1. This is exactly what the Revised Hierarchical Model [9] predicts. In terms of the level of processing framework [48], participants encoded the items at the semantic/conceptual level, gaining fast and accurate access to the conceptual representation that would provide information on such semantic features such as animacy, edibility, and so on. At the behavioral level this can lead to the two effects we have observed: faster picture-word association at the lexical level and better performance at the sentence level.

The present study leaves open some issues that can be dealt with in in the future. In particular, it would be useful to systematically manipulate the level of L2 proficiency in order to estimate the specific effect at each proficiency level identified, controlling for the impact of other cognitive factors, as well as to have stricter performance data available for the groups taking part to the study prior to the stat of the intervention stage. Also, a further interventional experimental group engaged in e.g. listening to music, may be of help to disentangle the effect of motor activity from e.g. motivation or interest brought about by the non-standard learning situation.

To conclude, our study shows evidence that physical activity improves L2 learning not only at the specific level of training (i.e., lexical level), but also at a more general, untrained level of processing (i.e., sentence level). Moreover, not only naive but also learners already exposed to the L2 may benefit from concurrent physical activity, whose effects were present even when tested after a month. This has relevant implication for the use of learning-supportive environments and, more in general, for theories of foreign language teaching.
